# Mothers' symptoms of anxiety and depression and the development of child temperament: A genetically informative, longitudinal investigation

**DOI:** 10.1002/jcv2.12171

**Published:** 2023-06-13

**Authors:** Y. I. Ahmadzadeh, E. M. Eilertsen, R. Cheesman, C. Rayner, E. Ystrom, L. J. Hannigan, T. A. McAdams

**Affiliations:** ^1^ SGDP Centre King's College London London UK; ^2^ PROMENTA Research Center University of Oslo Oslo Norway; ^3^ Centre for Fertility and Health Norwegian Institute of Public Health Oslo Norway; ^4^ School of Pharmacy University of Oslo Oslo Norway; ^5^ Nic Waals Institute Lovisenberg Diaconal Hospital Oslo Norway; ^6^ Department of Mental Disorders Norwegian Institute of Public Health Oslo Norway; ^7^ MRC Integrative Epidemiology Unit University of Bristol Bristol UK

**Keywords:** anxiety, depression, development, genetics, MoBa, temperament

## Abstract

**Background:**

Child temperament traits and mothers' emotional symptoms relating to anxiety and depression may drive changes in one another, leading to their ‘co‐development’ across time. Alternatively, links between mother and child traits may be attributable to shared genetic propensities. We explored longitudinal associations between mothers' emotional symptoms and child temperament traits and adjusted for genetic effects shared across generations.

**Methods:**

This study is based on the Norwegian Mother, Father and Child Cohort Study (MoBa). Mothers (*n* = 34,060) reported on their symptoms of anxiety and depression, and temperament among offspring (*n* = 42,526), at child ages 1.5, 3 and 5 years. Structural equation models parameterised developmental change in traits, and an extended family design adjusted for genetic effects.

**Results:**

We found individual differences in stable trait scores and rate of change for all study variables. Longitudinal *stability* in mothers' emotional symptoms was associated with longitudinal *stability* in offspring emotionality (*r* = 0.143), shyness (*r* = 0.031), and sociability (*r* = −0.015). Longitudinal *change* in mothers' symptoms showed very small or negligible correlations with longitudinal *change* in child temperament. Both genetic and environmental influences explained the stable longitudinal association between mothers' symptoms and child emotionality.

**Conclusions:**

The studied associations between mother and child traits across time appeared to be due to stable, trait‐like factors, involving genetic and environmental influence, rather than their co‐development. Findings contribute knowledge on how emotional symptoms develop in families across time, and the methods with which we can explore such development.

## Graphical abstract

We combine developmental modelling of longitudinal data with a genetically informative intergenerational design, to examine associations between child temperament and mothers' emotional symptoms relating to anxiety and depression. Findings contribute knowledge on how emotional symptoms develop in families across time, and the methods with which we can explore such development.

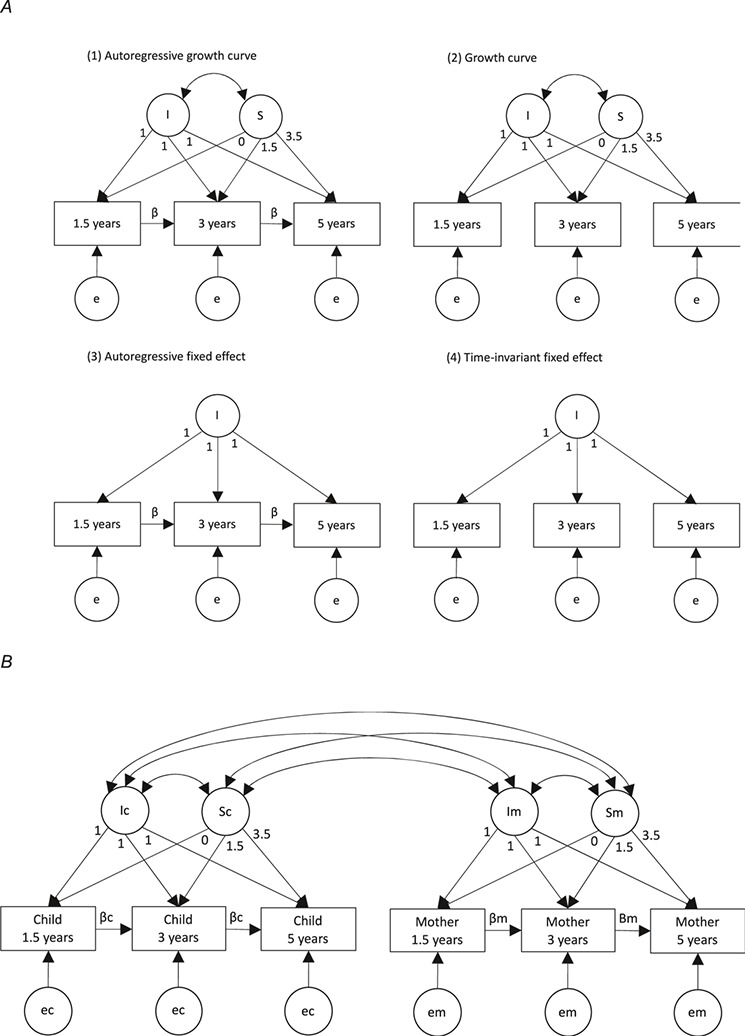

**Key points**

- We combined developmental modelling with a genetically informative design, to explore the nature of associations between early child temperament and mothers' emotional symptoms relating to anxiety and depression.
- In over 34,000 Norwegian families, mothers' emotional symptoms were weakly associated with child emotionality, shyness, and sociability—but not with child activity level—in the first 5 years of life.
- There was a stable element to the associations between mothers' emotional symptoms and child emotionality, shyness, and sociability across time. We showed that stable genetic and environmental factors linked mothers' symptoms and child emotionality (this could not be tested for other child temperament traits due to low intergenerational correlations).
- When we examined the rate of change in these variables across time, we found minimal evidence to suggest that mother's emotional symptoms change, or co‐develop, with child emotionality, shyness, and sociability across time.
- Researchers must continue to explore the origins of mental health in a developmental context, whilst adjusting for possible genetic influence.

## INTRODUCTION

Emotional symptoms relating to anxiety and depression are known to run in families (Connell & Goodman, 2002; Goodman et al., 2011; Kessler et al., 2007; Lawrence et al., 2018; Micco et al., 2009). During early child development, mothers' symptoms of anxiety and depression correlate with many child temperament traits (Britton, 2011; Hanington et al., 2010; Henrichs et al., 2009; McGrath et al., 2008; Melchior et al., 2012; Tees et al., 2010; Tronick & Reck, 2009). Further, extreme temperament scores are often associated with higher risk for emotional problems, in both cross‐sectional and prospective analyses from infancy through to adolescence (Abulizi et al., 2017; Goldsmith & Lemery, 2000; Goodyer et al., 1993; Martin et al., 2000; Rettew & McKee, 2005). For example, emotional temperament is shown to correlate with symptoms of anxiety, depression, conduct disorder, attention deficit hyperactivity disorder, and disordered eating during childhood. Understanding the mechanisms underlying associations between parent emotional symptoms and early child temperament could pave the way for better understanding of how emotional problems develop.

Measures of child temperament capture individual differences in reactivity (responsiveness to change in environments) and executive control (relating to physiological, affective and behavioural responses; Rothbart & Bates, 2006). Child temperament can be separated into numerous components, typically concerning individual differences in activity, withdrawal and affect (Rettew & McKee, 2005), which become evident within the first weeks of life (Rothbart et al., 2001; Zentner & Bates, 2008). Temperament traits are measured along continua, with extreme scores reflecting more ‘difficult’ temperament relative to the developmental period, for example, irregular bodily functions, withdrawal from new situations, slow adaptability, negative mood, or intense reactions in response to change.

While associations between child temperament and parent emotional symptoms are well‐documented, questions remain as to whether these associations are causal. Causal pathways are present if parents' symptoms directly influence the developmental course of offspring temperament, for example, via parenting styles and child social learning (Aktar et al., 2017; Lieb et al., 2000). Causal effects can also operate in the reverse direction, if child temperament influences parents' symptoms, for example, if difficult child temperament evokes parent stress (Kiff et al., 2011; Moe et al., 2018; Papousek & von Hofacker, 1998). However, adult emotional symptoms and child temperament, like nearly all human behavioural traits, are under genetic influence. This genetic influence comprises the effects of many genetic variants, each of which associate with many different traits (Goldsmith & Lemery, 2000; Levey et al., 2021; Nivard et al., 2015; Plomin et al., 2016; Saudino et al., 1998). This means that associations between parent emotional symptoms and child temperament may be attributable to influence from the same genetic variants, which the parent and child share by being related. In other words, genetic relatedness in families may lead to non‐causal (i.e., confounded) associations between parents' emotional symptoms and child temperament. To help us better understand the casual pathways underpinning parent‐offspring associations, researchers must consider and adjust for genetic influence.

Adoption, sibling‐comparison, and extended family designs allow researchers to adjust for the influence of genes shared between parents and children in intergenerational research. These designs enable identification of any non‐genetic, potentially causal, influences between generations. Evidence for non‐genetic associations between parent emotional symptoms and child temperament traits have been found in several adoption studies (Leve et al., 2019), for example, between parents' anxiety symptoms and infant negative affect, in genetically unrelated parent‐infant dyads (Brooker et al., 2015). However, generalisability of these findings to general populations remains a concern (Natsuaki et al., 2019). Research on exposure to parents' emotional symptoms in more representative samples using sibling‐comparison and extended family designs has mostly revolved around child psychopathology outcomes. Here, cross‐sectional parent‐child associations appear at least partly underpinned by non‐genetic pathways (Jami et al., 2021; McAdams et al., 2014). However, existing research is not sufficient for informing on longitudinal processes. Previous genetically informative studies have indexed developmental phenotypes using single assessments that are fixed in time. The possibility of *co‐development* between parent and offspring traits has not been considered.

Mothers' emotional symptoms and child temperament traits are subject to change across time, particularly during early child development, in the context of extensive social and physical development. Some mothers may experience an increase in feelings of anxiety or depression after the birth of a child, while others may experience the reverse, or no change at all (Ahmed et al., 2019; Campbell et al., 2007). Although long‐term stability in adult emotional symptoms is predominantly explained by genetic influence, change across time is best explained by transient environmental influence (Nivard et al., 2015; Torvik et al., 2019). Similar phenomena are observed for child temperament traits, where developmental change predominates over stability, particularly during early life with the honing of regulatory skills, and environmental influences explain variance in this change (Partridge & Lerner, 2007; Saudino et al., 1996). As such, environmental exposure to mothers' emotional symptoms could be driving *change* in offspring temperament traits across time, and vice versa. In one study, severity of depression symptoms in mothers was associated with a steeper increase in infant fearfulness, although the authors did not adjust for genetic influences shared between mothers and infants (Gartstein et al., 2010). To our knowledge, developmental pathways have not yet been explored in an intergenerational, genetically informative framework.

We investigated whether mothers' symptoms of anxiety and depression (herein referred to as emotional symptoms) co‐develop with offspring temperament traits across time, and whether links between these variables are best explained by genetic and/or causal influence. First, we used longitudinal structural equation models to explore the independent development of mother and child traits across time. Second, we combined longitudinal models across generations to explore co‐development of mother and child traits. Third, we use an extended family design to adjust for any genetic influence on intergenerational associations across time.

## METHODS

### Participants

Data were drawn from the Norwegian Mother, Father and Child Cohort Study (MoBa; Magnus et al., 2016). All pregnant mothers attending a routine ultrasound examination in Norway between 1999 and 2009 were invited to participate. Forty‐one percentage of eligible women accepted, yielding a total sample of >95,000 mothers, >75,000 fathers and >114,500 children. The establishment of MoBa and initial data collection was based on a license from the Norwegian Data Protection Agency and approval from The Regional Committees for Medical and Health Research Ethics. The MoBa cohort is currently regulated by the Norwegian Health Registry Act. Data are not available on participants' racial or ethnic identity, although data from Statistics Norway suggest that approximately 90% of the Norwegian population had Norwegian‐born parents and grandparents the year that MoBa recruitment ended (Statistics Norway, 2020).

Current analyses are based on the Intergenerational Transmission of Risk subproject, where MoBa data are linked with pedigree and zygosity information from the Medical Birth Registry of Norway and the Norwegian Twin Registry. Here we identified pedigree structures within the MoBa dataset, building a sample comprising extended family units of adult siblings (twins, full‐siblings, or half‐siblings) or first cousins, and their children. Within each extended family unit, we extracted available data from up to two mothers (i.e., adult females matched with their sibling or first cousin, or their spouse's sibling or first cousin), with up to two children per mother. Some mothers in the MoBa dataset do not have extended family members taking part in the study. These mothers were included as nuclear family units if they had two children in the study. Phenotypic data were drawn from version 12 of the MoBa quality‐assured data files. In total, raw data were used for 42,526 offspring born to 34,060 mothers. Not all mothers participated at every age and attrition was evident over time (Ns for each assessment were as follows: 1.5 years = 38,939 children to 31,567 mothers; 3 years = 31,704 children to 25,976 mothers; 5 years = 23,450 children to 20,032 mothers). Table 1 provides an overview of the pedigree structures extracted for the genetically informed, extended family design.

**TABLE 1:** Overview of pedigree structures used for biometric Multiple‐Children‐of‐Twins/Siblings model analyses.

Extended families (*N* = 20,921)	*r*A	*n*
*N* stratified by the parent pairs used to identify extended families^a^		
Identical twin pair	1.00	67
Full‐sibling or fraternal twin pair	0.500	13,147
Maternal/paternal half‐sibling pair	0.250	754
Cousin pair	0.125	6953
*N* stratified by mothers' relatedness in each extended family^b^		
Identical twin pair	1.00	48
Full‐sibling or fraternal twin pair	0.500	4407
Maternal/paternal half‐sibling pair	0.250	290
First cousin pair	0.125	2972
Unrelated sisters/cousins‐in‐law pair	0.000	13,204
Number of offspring pairs linked to each mother		
Full‐sibling pair	0.500	5579
Maternal half‐sibling pair	0.250	49
Unpaired (single) offspring	–	23,314

Unpaired nuclear families (*N* = 5083)^c^		
Number of offspring pairs linked to each mother		
Identical twin pair	1.00	111
Full‐sibling or fraternal twin pair	0.500	4951
Maternal half‐sibling pair	0.250	21

*Note*: Numbers represent families who had data for at least one individual in our biometric analyses.

Abbreviation: *r*A, genetic relatedness coefficient.

^a^ Extended families were identified via adult twin, sibling or cousin pairs enroled in the MoBa cohort as parents. These included mother‐mother, mother‐father, or father‐father pairs.

^b^ Phenotypic data were derived from mothers in each extended family. Mothers were genetically unrelated (*r*A = 0.000) if their extended family was identified using a mother‐father or father‐father pair (i.e., mothers in these families were sister‐in‐law or cousin‐in‐law pairs).

^c^ Mothers who could not be paired into an extended family (i.e., because they did not have a twin, sibling, cousin, sister‐in‐law, or cousin‐in‐law in the dataset) were modelled independently in nuclear family units if they had more than one offspring. Mothers who had twin offspring were also modelled independently in nuclear family units.

### Measures

Mothers' emotional symptoms were measured by self‐report when children were aged 1.5, 3 and 5 years, using the eight‐item version of the short form Hopkins Symptom Checklist (SCL‐8; Hesbacher et al., 1980; Tambs & Røysamb, 2014). Mothers reported on the extent to which they experienced four symptoms relating to anxiety (e.g., nervousness or shakiness) and four symptoms of depression (e.g., feeling hopeless about the future), using a four‐point Likert scale (‘not bothered’ to ‘very bothered’). Mean scores were calculated for each time‐point, excluding participants who had missing data for more than half of the items. Internal consistency (Cronbach's alpha) was 0.85, 0.87 and 0.86 at each timepoint respectively.

Offspring temperament was rated by mothers at the same timepoints, using the Emotionality, Activity and Shyness Temperament Questionnaire (EAS; Mathiesen & Tambs, 1999). Twelve items assessed four temperament traits: emotionality (e.g., child gets upset or sad easily), activity level (e.g., child is always on the go), shyness (e.g., child takes a long time to warm up to strangers), and sociability (e.g., child finds other people more fun than anything else). Mothers responded using a five‐point Likert scale (‘very typical’ to ‘not at all typical’). Cronbach's alpha for each trait respectively were: 1.5 years 0.64/0.65/0.65/0.32, 3 years 0.64/0.64/0.66/0.51 and 5 years 0.75/0.75/0.70/0.71. One item indexing child sociability was not included in the 1.5‐year assessment. Mean scores for each temperament trait were calculated for each timepoint, excluding participants missing on more than half of the trait‐related items. Each trait was scaled such that higher scores indexed a stronger phenotype (e.g., maximum shyness score = maximum shyness; maximum sociability score = maximum sociability).

All study variables were regressed on covariates—maternal age, child year of birth, parity, and child sex—and the resultant residuals were standardised.

### Data modelling procedure

Our data modelling procedure followed a sequence of incremental steps: (i) modelling **longitudinal development** of each mother and child variable; (ii) modelling **co‐development** for each combination of mother‐child variables; (iii) modelling extended family data to **adjust for the role of genetics** in any longitudinal, cross‐generation associations. Step (iii) was taken only for associations greater than 0.1, to guard against loss of statistical power. All analyses were conducted in R (version 3.4.4). Steps (i) and (ii) used *lavaan* with Full Information Maximum Likelihood (version 0.6‐5; Rosseel, 2012). Step (iii) used *OpenMx* (version 2.12.1; Neale et al., 2016). Cluster‐robust standard errors were used to account for any effects of data structuring within family units (further detail in Supporting Information S1: Appendix S1).

#### Longitudinal development of each mother and child variable

**FIGURE 1:**
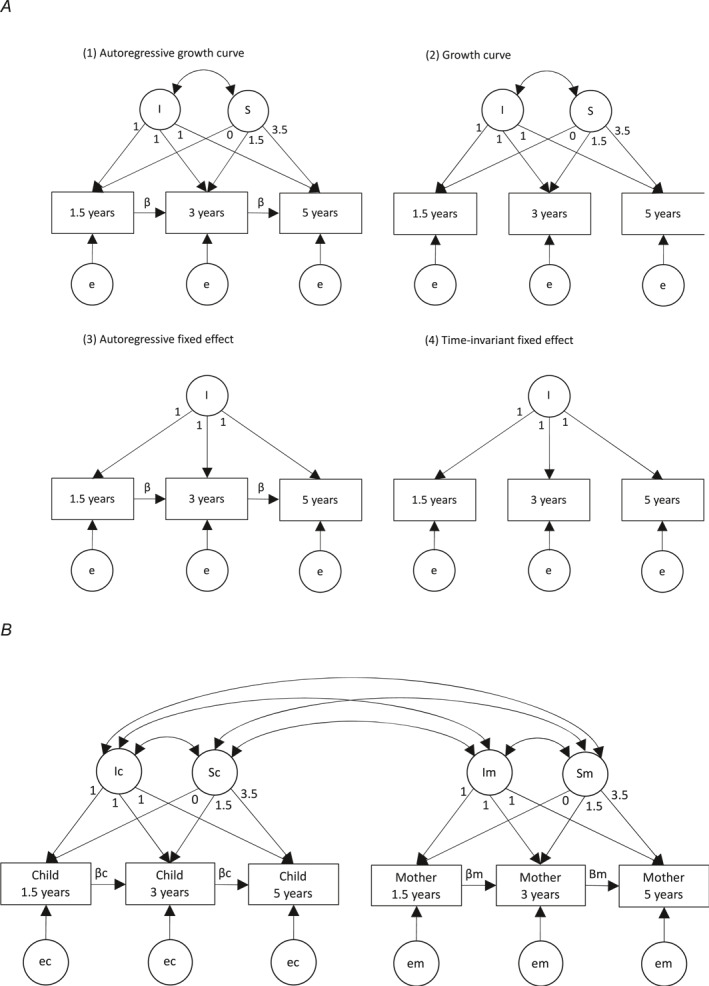
Structural equation models for parameterising developmental change. (A) Models fitted to explore the longitudinal development of each mother and child variable. (B) Model fitted to explore the longitudinal co‐development for each combination of mother‐child variables. Rectangles denote measured variables. Circles denote latent factors. One‐headed arrows denote regression paths. Double‐headed arrows denote correlations. β, autoregressive paths (capturing within‐person, longitudinal prediction of each variable on itself); c, child; e, residual error (capturing time‐variant variance, set to be equal across observations for each variable); I, intercept (capturing time‐invariant variance); m, mother; S, slope (capturing linear change across observations from 1.5 years). Panel A shows models used to explore trait cross‐time covariance structure. Models 2–4 are nested in Model 1. Panel B shows an example of a model used to explore cross‐generation, cross‐time covariance structure of mother and child traits (applying the autoregressive growth curve model in both generations).

We fitted autoregressive growth curve models to explore longitudinal change in each mother and child variable across time (Bollen & Curran, 2004). Model parameters are depicted in Figure 1A (Model 1). Here, the latent intercept factor (I) captured stable variance at 1.5 years plus any persistent, stable variance shared at 3 and 5 years for each variable. The latent slope factor (S) captured variance in the rate at which each variable changed linearly from the first assessment across time (i.e., loading = 0 at 1.5 years). The residual error factors (e) captured unique variance at each time point. The autoregressive paths (*β*) captured longitudinal prediction of each variable on itself over time, providing an association between the error terms. Error factors and autoregressive paths were constrained to be time invariant, to ensure model identification and prevent over‐fitting.

Autoregressive growth curve models were trimmed to test whether the latent slope factor and/or autoregressive paths (i.e., parameters that index developmental change) could be dropped without significant loss of model fit for each variable. Figure 1A depicts the three trimmed (i.e., nested) models tested for each variable (models 2–4). Model 4 was the simplest, most parsimonious model, indexing a purely stable, persistent variable structure across time (i.e., a time‐invariant fixed effect model).

Chi square goodness of fit tests were used to statistically compare the fit of models 2–4 to that of model 1 (further detail in Supporting Information S1: Appendix S1). The best fitting model for each variable was taken forward for analyses exploring co‐development across generations.

#### Co‐development for each combination of mother‐child variables

We combined the best fitting model for development of mothers' emotional symptoms with the best fitting model for development of each child temperament trait, to explore co‐development across generations. Here, latent slope and intercept factors for each variable were allowed to correlate across generations. Figure 1B shows model specification for the cross‐generation autoregressive growth curve model.

Cross‐generation models were then trimmed to test whether the cross‐generation latent factor correlations could be dropped without significant loss of model fit, again using Chi square goodness of fit tests to compare nested models to the full model. If dropping a cross‐generation latent factor correlation compromised model fit, then evidence for a significant longitudinal intergenerational association was found. Significant correlations between latent slope factors would indicate co‐development of traits. For any longitudinal cross‐generation associations greater than 0.1, latent factor scores were extracted and taken forward for genetically sensitive analyses using the extended family design.

#### Adjusting for genetic effects

We used an extended family design to separate the role of genetic versus environmental pathways underpinning significant mother‐child latent factor correlations (i.e., longitudinal intergenerational associations). We fitted a Multiple‐Children‐of‐Twins/Siblings model (MCoTS, full path diagram in Supporting Information S1: Figure S1; McAdams et al., 2018) for correlations greater than 0.1 (covariance decompositions are less informative for weak associations as demands on statistical power increase; Ahmadzadeh et al., 2021). MCoTS models are an extension of the traditional twin model (Plomin et al., 2012), as detailed in Supporting Information S1: Appendix S2, requiring data from adult parents and their offspring in extended families. Results provide information on the genetic and environmental influences on parent traits, child traits, and the covariance between these traits in the given sample.

## RESULTS

Descriptive statistics for the raw study data are shown in Supporting Information S1: Table S1. Total scores for mothers' emotional symptoms were low on average, though positively skewed distributions show that the sample includes mothers with emotional difficulties. Child temperament scores were moderate on average and normally distributed. Cross‐sectional correlations between mother‐offspring variables are shown in Table 2.

**TABLE 2:** Phenotypic, cross‐sectional correlations between mothers' emotional symptoms and child temperament traits during early childhood.

Child temperament	Mother emotional symptoms		
	1.5 years	3 years	5 years
Emotionality	0.15*	0.15*	0.18*
Activity level	−0.01	−0.01	0.02
Shyness	0.03*	0.04*	0.05*
Sociability	−0.02	−0.03*	−0.04*

*p < 0.001, using Bonferroni correction to adjust for multiple tests.

Cross‐sectionally, mothers' emotional symptoms were most strongly linked to child emotional temperament (*r* = 0.15–0.18) and were very weakly linked to child shyness and sociability (*r* ≤ 0.05). Child activity level was not associated with mothers' emotional symptoms at any timepoint in our sample (*r* ≤ 0.02) and was therefore not carried forward for analysis.

### Longitudinal development of mother and child variables

Mother and child data were first analysed separately. Four longitudinal models were fit sequentially to each variable (Figure 1; fit statistics in Supporting Information S1: Table S2). The autoregressive growth‐curve model (Figure 1A, Model 1) fit the data best for all variables (Supporting Information S1: Table S3), showing that mothers' emotional symptoms and all child temperament traits could be characterised by: (1) model **intercepts**, indexing stability in the variable across time; (2) model **slopes**, indexing rate of linear change in the variable across time; (3) residual **error factors**, indexing variance specific to each time point; and (4) **autoregressive paths**, indexing the lagged prediction of each variable on itself over time.

For each variable, variance in the intercept factor was substantial, highlighting individual differences in the stable component of participants' trait scores (0.279–0.554; Table 3). In contrast, the slope factors for each variable had little variance, suggesting that the rate of change over time did not differ much between participants (0.005–0.018; Table 3). For offspring sociability, the slope factor variance was not significant and was thus fixed to zero without compromising model fit (Supporting Information S1: Table S4). Weak, negative correlations were found between intercept and slope factors for each variable, indicating that participants who started with higher trait scores saw a greater reduction in their score across time, and vice versa (*r* = −0.041–−0.013; Table 3; consistent with regression to the mean). Significant autoregressive paths (*β* = 0.068–0.113; Table 3) showed that participants' scores at one time point had a lagged effect on their score at the subsequent time point. For each variable, the autoregressive growth‐curve model was carried forwards for inclusion in cross‐generation longitudinal models.

**TABLE 3:** Parameter estimates (95% confidence intervals) in each within‐generation autoregressive growth‐curve model.

	Variance in stability: I	Variance in rate of linear change: S	Correlation (*r*): I–S	Autoregressive effects: *β*	Residual error: e
Child emotionality	0.434	0.014	−0.035	0.097	0.559
	(0.408, 0.460)	(0.011, 0.017)	(−0.040, −0.029)	(0.075, 0.119)	(0.540, 0.578)
Child activity	0.492	0.018	−0.041	0.106	0.493
	(0.468, 0.516)	(0.015, 0.021)	(−0.046, −0.037)	(0.085, 0.128)	(0.477, 0.509)
Child shyness	0.449	0.015	−0.035	0.113	0.530
	(0.422, 0.475)	(0.012, 0.018)	(−0.040, −0.030)	(0.090, 0.137)	(0.511, 0.548)
Child sociability	0.279	0.005	−0.013	0.112	0.697
	(0.245, 0.313)	(−0.000, 0.009)	(−0.019, −0.006)	(0.085, 0.140)	(0.670, 0.724)
Mother emotional symptoms	0.554	0.012	−0.032	0.068	0.460
	(0.533, 0.575)	(0.009, 0.014)	(−0.036, −0.027)	(0.049, 0.087)	(0.447, 0.472)

Abbreviations: I, latent intercept factor; S, latent slope factor.

### Co‐development of mother and child variables

A cross‐generation autoregressive growth‐curve model (i.e., Figure 1B) fit the data well for all mother‐child variable combinations (fit statistics in Supporting Information S1: Table S2). Cross‐generation latent factor correlations are shown in Table 4 (grey boxes) and non‐significant correlations could be dropped without compromising model fit (Supporting Information S1: Table S5). Results showed that the intergenerational correlations were predominantly explained via their stable factors across time, and to a very small degree, if not negligible, by their co‐development across time.

**TABLE 4:** Correlation matrix for latent factors in each cross‐generation autoregressive growth‐curve model.

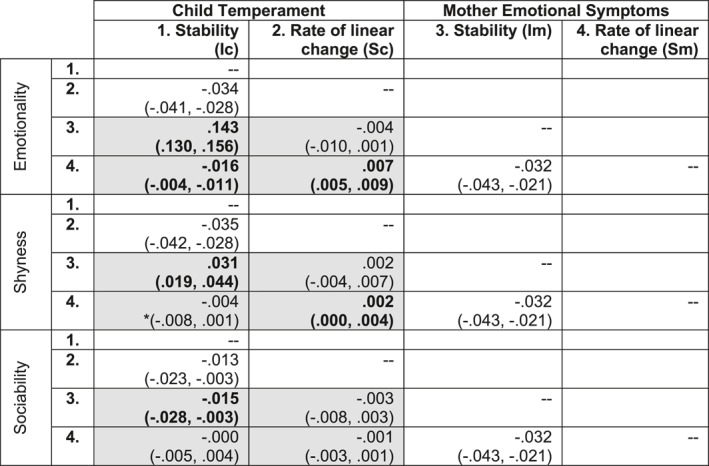

*Note*: White boxes, within‐generation latent factor correlations. Grey boxes, cross‐generation latent factor correlations. Bold, parameters that could not be dropped from the model without significant detriment to model fit. Confidence intervals are Bonferroni adjusted to account for four multiple tests with mother symptoms (i.e., intergenerational associations with mothers' emotional symptoms were examined four times, using each child temperament trait, so confidence intervals were corrected to 98.75% [1 − (0.05/4)]). One parameter became non‐significant after Bonferroni adjustment (*).

Abbreviations: c, child; I, latent intercept factor; m, mother; S, latent slope factor.

The strongest link was between stable trait scores (as indexed by latent intercept factors) for mothers' emotional symptoms and offspring emotionality (*r* = 0.143). Mothers' stable trait scores were also correlated with stable trait scores for child shyness (*r* = 0.031), and child sociability (*r* = −0.015). Intergenerational correlations involving rate of linear change in mother and child traits (as indexed by latent slope factors) were all very small (*r* < 0.005), showing minimal evidence for co‐development of mothers' emotional symptoms and any of the child temperament traits. However, rate of linear change in mothers' emotional symptoms were negatively correlated with stable trait scores in child emotional temperament (*r* = −0.016), which was the greatest intergenerational correlation involving a latent slope factor.

### Adjusting for genetic effects

Latent factor scores were extracted from the *within‐generation* autoregressive growth curve models for use in the genetically informative extended family models. Only the association between stable maternal emotional symptoms and stable offspring emotionality (*r* = 0.191) met our pre‐specified threshold for inclusion in the genetic decomposition analyses (*r* > 0.1). We used an extended family model (Supporting Information S1: Figure S1) to reveal the relative contribution of genetic and environmental causes underpinning the association between stable trait scores in mothers' emotional symptoms and offspring emotionality across time. Parameter estimates for the base model are shown in Supporting Information S1: Figure S2, which was subsequently trimmed to maximise model parsimony (removing parameters C1 and c1’; Supporting Information S1: Table S6).

**FIGURE 2:**
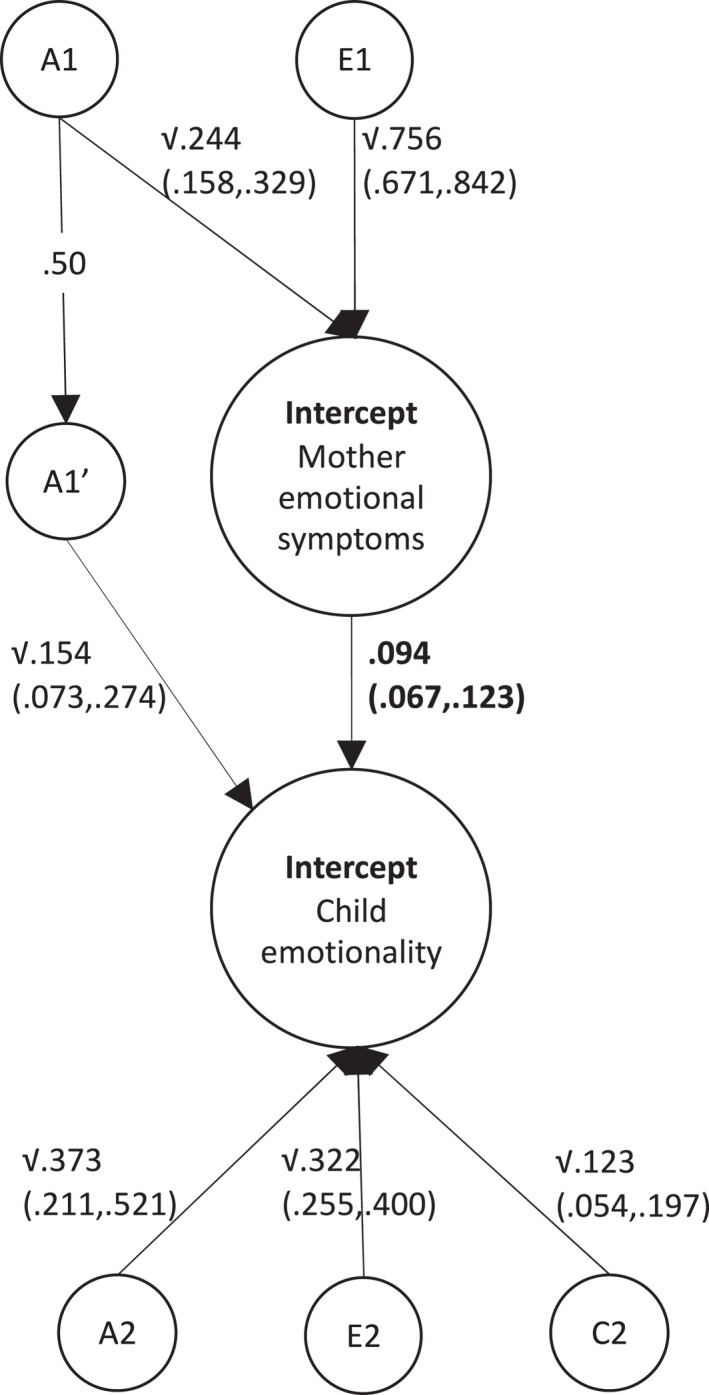
Results from Multiple‐Children‐of‐Twins/Siblings model biometric analyses, decomposing the association between stability in mothers' emotional symptoms and stability in child emotionality (*r* = 0.191). A1, additive genetic effects on mother trait; A2, additive genetic effects specific to child trait; A1′, additive genetic effects common to mother and child traits (path from A1 to A1′ is fixed to 0.50 because offspring inherit 50% of their mother's genes); C2, common environment effects on child trait; E1/E2, unique environment effects on mother/child trait. Variance components are displayed in non‐bold text. The residual intergenerational association is displayed as a standardised path beta coefficient in bold text. Figure represents a partial path diagram.

The total correlation (*r* = 0.191) between stable trait scores for mothers' emotional symptoms and offspring emotionality could be partitioned into two equal components. Half of the association was accounted for by mother‐offspring genetic relatedness (50.7%, CI 36.1%–64.6%).^1^ The other half was accounted for by environmentally mediated, direct effects (49.3%, CI 35.4%–63.9%). Both contributions were significant, neither the genetic nor environmental effects could be dropped without significant deterioration in model fit (Supporting Information S1: Table S6). Model results are shown in Figure 2 and a more detailed description of these results is provided in Supporting Information S1: Supplementary Text 2.

## DISCUSSION

We set out to combine developmental modelling of longitudinal data with a genetically informative intergenerational design, to examine associations between child temperament and mothers' emotional symptoms relating to anxiety and depression. In over 34,000 families in Norway, we observed weak correlations between mothers' emotional symptoms and child emotionality, shyness, and sociability—and no association with child activity level—in the first 5 years of life. We showed that these weak intergenerational correlations were predominantly explained by their stable factors across time (i.e., latent intercept factors for the mother and child variables were correlated). Correlations between ‘change over time’ in mothers' symptoms and child temperament were negligible (i.e., latent slope factors for the mother and child variables were very weakly correlated, if at all). In other words, although these mother and child traits appeared to share stable influences across time, they did not appear to co‐develop across time in early childhood. Finally, we showed that the correlation between mothers' stable emotional symptoms and offspring stable emotional temperament was influenced by both genetic and environmental factors shared between mother and child. Genetically informed analyses could not be conducted for the other child temperament traits due to their low correlations with mothers' symptoms.

### Mothers' emotional symptoms were most linked to child emotional temperament

The observed cross‐sectional correlations between mothers' emotional symptoms and offspring emotionality in our dataset were consistent with previous research (e.g., Britton, 2011; McGrath et al., 2008; Tronick & Reck, 2009). These findings support the notion that mothers who self‐report higher levels of emotional symptoms are more likely to report higher levels of emotionality in their children. However, this pattern was weaker for child shyness and sociability, and not observed for child activity level. This contrasts with previous research showing larger, significant phenotypic correlations between mothers' emotional symptoms and a range of child temperament traits, including activity, affect, adaptability and approach (e.g., Britton, 2011; Henrichs et al., 2009; Tees et al., 2010). Publication bias could have contributed to the predominantly significant, positive results observed in the existing literature, and/or differences in measurement techniques or developmental period. Our data suggest that child temperament traits do not always correlate with mothers' emotional symptoms, although child emotional temperament may be a useful marker for the early detection of familial risk for emotional problems. Accordingly, previous research highlights child emotional temperament as a key predictor for child internalising and externalising psychopathology (Abulizi et al., 2017; Goodyer et al., 1993; Martin et al., 2000).

### Minimal evidence for co‐development of mothers' emotional symptoms and child temperament traits across time

We found minimal evidence to suggest that change in mothers' emotional symptoms was associated with change in child temperament. Previous twin studies suggest that change in adult emotional symptoms and child temperament is significantly influenced by environmental factors (Hannigan et al., 2016; Nivard et al., 2015; Saudino et al., 1996; Torvik et al., 2019). In our analyses, the environmental factors driving change in mothers' emotional symptoms were not indexed by change in child temperament, and vice versa. This may be reflective of the developmental period we studied, characterised by extreme child change between 1.5 and 5 years. Future research could explore parent‐child co‐development at later, relatively more stable developmental periods.

Previous research in the same Norwegian sample found no prospective prediction from mothers' emotional symptoms to offspring psychopathology symptoms during early child development, after correction for contemporaneous associations and genetic effects (Gjerde et al., 2017, 2018). Methodologically, we build on this work to explore developmental processes in the context of child temperament, examining correlations between latent factors that capture variance in each generation across multiple timepoints. However, all longitudinal approaches using data from the MoBa cohort rely upon temporally wide timeframes, with measures collected every 1.5–2 years. This restricts our ability to explore longitudinal processes within shorter windows, where results may differ.

### Genetic and environmental influences on the stable association between mothers' emotional symptoms and child emotional temperament

To our knowledge, this is the first study to demonstrate genetic overlap between adult emotional symptoms and early child emotional temperament. Approximately half of the stable, trait‐level association across time between mothers' emotional symptoms and offspring emotional temperament was attributable to their genetic relatedness. Similar results are observed in the same sample for cross‐sectional associations involving child psychopathology symptoms (Gjerde et al., 2017, 2018, 2019), which correlate with child temperament but do not share the same continuum (Rettew & McKee, 2005). In much smaller samples outside of Norway, researchers have observed no genetic overlap between adult emotional symptoms and child emotion‐related outcomes (Jami et al., 2021; McAdams et al., 2014), which could in part reflect their limited statistical power for detecting intergenerational genetic correlations (Ahmadzadeh et al., 2021). In our sample, results suggest that intervention approaches to support families affected by emotional symptoms should be designed with consideration for the role of stable genetic risk alongside social factors.

After adjusting for the role of genetics, the residual environmentally mediated effect between stable emotional symptoms in mothers and offspring was half of the original estimate (standardised *β* = 0.094, CI 0.067–0.123). This suggests that stable similarity in levels of emotional symptoms between mothers and offspring during early development can in part be influenced by social interactions between mother and child. These influences could potentially be negated by family‐level intervention, to prevent the perpetuation of emotional symptoms in families. Crucially, previous research shows such findings differ by child outcome (Jami et al., 2021; McAdams et al., 2014), emphasising the importance of considering our results only in the context of the traits examined. In this study we could not repeat our genetically informed analyses for other child temperament traits due to their low correlations with mothers' symptoms.

### Limitations

This study involved a sophisticated developmental and genetically informative research design in a large sample. These strengths notwithstanding, some methodological limitations require mention. First, all phenotypes were reported by mothers. Data collection from multiple reporters is logistically and financially difficult in large cohorts and mothers are paramount for informing on behaviours in young offspring. However, intergenerational correlations may have been inflated by shared rater bias. For example, mothers experiencing higher levels of emotional symptoms may be more inclined to perceive higher emotionality in their child. While this is a common limitation in family mental health research, it is important to note that our analyses did not rely entirely on mother‐child variable comparisons. Our extended family design also used avuncular correlations to derive intergenerational associations (i.e., comparing mothers' self‐reported emotional symptoms with their sister/cousin/sister‐in‐law's report of their niece/nephew's temperament), which were not inflated by shared rater bias. In an ideal design we would include additional data from co‐parents in the models.

Second, Norwegian mothers participating in our studied cohort experienced lower levels of emotional symptoms on average, compared to those who did not volunteer to participate from the Norwegian population, and compared to those who were lost to the study through attrition (Nilsen et al., 2009). However, previous work suggests that low prevalence rates do not necessarily lead to biased estimates of associations between exposures and outcomes (Nilsen et al., 2009). Third, data were not collected on the racial and ethnic identity of participants in our sample, although information from Statistics Norway suggests they would identify predominantly as white European (Statistics Norway, 2020). As in all research, we urge caution in extrapolating our findings across populations with varied demographics and in different environments, including those with different racial and ethnic identities, levels of impairment, child age, parent sex and gender, geographical and national locations.

Fourth, analyses could be improved by use of more frequent longitudinal assessments, shedding light on how parents and offspring change within years, not just between years. Longer, more detailed measures may also help to identify more nuanced sources of parent‐offspring similarity. Collecting longer questionnaires and repeat data collections across shorter timeframes is a challenge when working with the sample sizes required for genetically informative research. Finally, our analyses required balance in maintaining model complexity, statistical power, and interpretability of results, in the context of expectedly small effect sizes between mother and child variables. We were required to make subjective decisions to balance these factors (e.g., which intergenerational correlations we take forward for genetically informed analyses), which is a common challenge in this field of research.

## CONCLUSION

In our sample, mothers' emotional symptoms relating to anxiety and depression were correlated with offspring emotionality, shyness, and sociability—but not activity level—in the first 5 years of life. These correlations appeared due to stable, trait‐like factors across time. For offspring emotional temperament, we uncovered both stable genetic and environmental links to mothers' emotional symptoms. Although longitudinal change was observed in mothers' symptoms and offspring temperament traits, we found no substantial evidence for their intergenerational co‐development across time. These findings help to build our understanding as to how emotional symptoms develop in families across time, and the methods with which we can explore such development.

## AUTHOR CONTRIBUTIONS

**Y. I. Ahmadzadeh**: Conceptualization; data curation; formal analysis; investigation; methodology; project administration; writing – original draft; writing – review & editing. **E. M. Eilertsen**: Data curation; formal analysis; methodology; software; writing – review & editing. **R. Cheesman**: Conceptualization; investigation; writing – review & editing. **C. Rayner**: Writing – review & editing. **E. Ystrom**: Conceptualization; data curation; formal analysis; funding acquisition; investigation; methodology; project administration; resources; software; supervision; writing – review & editing. **L. J. Hannigan**: Conceptualization; data curation; formal analysis; investigation; methodology; supervision; writing – review & editing. **T. A. McAdams**: Conceptualization; data curation; formal analysis; funding acquisition; investigation; methodology; project administration; resources; software; supervision; writing – review & editing.

## CONFLICT OF INTEREST STATEMENT

Eivind Ystrom is a Joint Editor for JCPP Advances. Tom McAdams serves on the JCPP Advances Editorial Advisory Board. The remaining authors have declared that they have no competing or potential conflicts of interest.

## Ethical considerations

The establishment of MoBa and initial data collection was based on a license from the Norwegian Data Protection Agency and approval from The Regional Committees for Medical and Health Research Ethics.

## Supporting information

Supporting Information S1

## Data Availability

The data that support the findings of this study are from The Norwegian Mother, Father and Child Cohort Study (MoBa). Restrictions apply to the availability of these data, which were used under license for this study.

## ENDNOTE

^1^
The relative contribution of genetic relatedness (A1′ in Figure 2) in explaining the parent‐child correlation is calculated by multiplying the genetic paths connecting each trait, divided by the total phenotypic correlation. According to results in Figure 2, this calculation is: ([√0.224 × 0.50 × √0.154]/0.191).
