# Supplementary material for: Mothers' symptoms of anxiety and depression and the development of child temperament: A genetically informative, longitudinal investigation
Source: JCPP Adv. 2023 Jun 13;3(4):e12171. doi: 10.1002/jcv2.12171 (PMC10694534; doi:10.1002/jcv2.12171)
Supplement: Supplementary file 1 — Supporting Information S1 [file JCV2-3-e12171-s001.docx]

**Mothers’ symptoms of anxiety and depression and the development of child temperament: A genetically informative, longitudinal investigation**

Dr Y. Ahmadzadeh, Dr E.M. Eilertsen, Dr R. Cheesman, Dr C. Rayner, Dr E. Ystrom, Dr L. J. Hannigan, Dr T. A. McAdams

Supplementary materials

- **Supplementary Text 1.** Notes on model fitting for longitudinal development and developmental interplay.
- **Supplementary Text 2.** Notes on model fitting for the Multiple-Children-of-Twins/Siblings (MCoTS) model, used to adjust for genetic effects
- **Table S1.** Descriptive statistics for raw data measured using the Emotionality, Activity and Shyness Temperament Questionnaire for offspring and the short form of the Hopkins Symptom Checklist for mothers.
- **Table S2.** Model fit statistics for within- and cross-generation autoregressive growth-curve models
- **Table S3.** Chi square goodness of fit tests for longitudinal modelling of mother and child variables
- **Table S4.** Goodness of fit tests and parameter estimates for the autoregressive growth curve model (Figure 1, Model 1) fit to within-generation child sociability data: fixing variance in the latent slope factor to zero.
- **Table S5.** Chi square goodness of fit tests for nested models in longitudinal analyses of phenotypic, cross-generation data: dropping intergenerational latent factor correlations
- **Table S6.** Model fit statistics and comparisons for MCoTS models decomposing the association between stability in mothers’ emotional symptoms and stability in child emotionality
- **Figure S1.** Full MCoTS structural equation model, based around pairs of mothers who are twins, full-siblings, half-siblings, cousins or unrelated sisters-in-law
- **Figure S2.** Base model results from MCoTS biometric analyses, decomposing the association between stability in mothers’ emotional symptoms and stability in child emotionality

**Supplementary Text 1. Notes on model fitting for longitudinal development and developmental interplay**

***Exploring possible effects of the nested data structure***

Cluster-robust standard errors were used to account for any effects of structuring of the data in nuclear family units (i.e., non-independence of data points collected from the same mother). If results from robust and classical standard errors diverged then we planned to use multilevel approaches to examine the effects of nested data structures (King & Roberts, 2015). This was not necessary, given that analyses using classical standard errors did not diverge from results using cluster-robust standard errors, thereby suggesting no effect of the nested data structure on results.

***Chi square goodness of fit tests***

Chi square goodness of fit tests were used to statistically compare the fit of models 2 – 4 to that of model 1 (see models in manuscript Figure 1). If a nested model (2 – 4) was found to maintain model fit in comparison to model 1, then this model would be selected for use in further analyses. If more than one nested model was found to maintain model fit in comparison to model 1, then these nested models were compared with one another using further Chi square tests (i.e., if the nested models were also nested within one another: as for Model 4 in 2 or 3) or by visual inspection of the fit statistics (i.e., if nested models were not nested within one another: as for Models 2 and 3). Visual inspection of the fit statistics would involve comparing Akaike Information Criterion (AIC) and Bayesian Information Criterion (BIC) statistics (see Table 3) for each model, with lower values indicating better model fit.

***References***

King, G., & Roberts, M. E. (2015). How Robust Standard Errors Expose Methodological Problems They Do Not Fix, and What to Do About It. *Political Analysis, 23*(2), 159-179. doi:10.1093/pan/mpu015

**Supplementary Text 2. Notes on model fitting for the Multiple-Children-of-Twins/Siblings (MCoTS) model, used to adjust for genetic effects**

***Understanding the logic behind the model***

MCoTS models require data from adult parents and their offspring in extended families, to create a natural quasi-experiment. When identical twins have children, their offspring are just as genetically related to their own parent compared to their parent’s twin (genetic correlation=.50). When fraternal twins or full-siblings have children, their offspring are more genetically related to their own parent (genetic correlation=.50) than their parent’s twin or sibling (genetic correlation=.25). Accordingly, the offspring of half-siblings and cousins are more genetically related to their own parent than to their parent’s half-sibling or cousin. Alongside genetic relatedness estimates, it is assumed that all children share their immediate rearing environment only with their parent, not with their parent’s sibling or cousin. Trait associations between children and their parent’s sibling or cousin (referred to as ‘avuncular’ associations)^[[1]](#footnote-1)^ can then be used to estimate genetic effects on parent-child associations. If avuncular associations are stronger in extended families who are more genetically related, this indicates a role of shared genetics acting across generations. Once the role of shared genetics is accounted for, any residual intergenerational association is attributable to influence in the family environment, plus error. Following this logic, MCoTS models provide three sets of information, outlined below in the context of this study.

First, data from pairs of differentially related mothers (i.e., twins, full- and half-siblings, cousins, and unrelated sisters/cousins-in-law) are used to decompose variance in mothers’ emotional symptoms intro three latent factors: population variance explained by additive genetic influences (A1), common environmental influences that make individuals more similar (C1), or unique environmental influences that make individuals more different (E1) (as in the traditional twin design; Plomin et al., 2012). Second, data from differentially related children (i.e., twins, full-siblings, maternal-half-siblings, and cousins) are used to decompose variance in child temperament into equivalent genetic and environmental components (A2, C2, and E2). Third, data from differentially related avuncular pairs are used to decompose covariance between generations into three paths: effects of genetic transmission (a1’; accounting for the effects of genes shared between generations); effects of the extended family environment (c1’; accounting for the effects of environments that are shared between all members of an extended family); and residual phenotypic transmission (*p*; accounting for environmental effects shared between the parent and child, plus any sources of confounding that remain unaccounted for) (see Figure S1).

***Interpreting the model results***

In the parsimonious model (Figure 2), 22.4% (CI 15.8 – 32.9%) of the variance in stable trait scores for mothers’ emotional symptoms could be explained by mothers’ genetics (A1). In total, 52.7% of the variance in stable trait scores for child emotionality could be explained by child genetics (A1’+A2=37.3%+15.4%). Specifically, 37.3% (CI 21.1 – 52.1%) of the child variance was explained by genetic factors unique to the child generation (A2), while 15.4% (CI 7.3 – 27.4%) was explained by shared genetic factors influencing both child and mothers’ emotional symptoms (A1’). This meant that 29% of the genetic variants influencing childhood emotionality were associated with those influencing emotional symptoms in adult mothers (A1’/(A1’+A2)=15.4/52.7). After the role of genetics was accounted for, we observed a significant residual association between stable trait scores both for mothers’ emotional symptoms and offspring emotionality (β=.094, CI .067 - .123).

**Table S1. Descriptive statistics for raw data measured using the Emotionality, Activity and Shyness Temperament Questionnaire for offspring and the short form of the Hopkins Symptom Checklist for mothers.**

|  | Age | n | Mean | SD | Skew | Kurtosis |
| --- | --- | --- | --- | --- | --- | --- |
| Child emotionality | 1.5 | 38676 | 2.73 | .76 | .10 | -.12 |
|  | 3 | 31601 | 2.79 | .77 | .11 | -.10 |
|  | 5 | 23354 | 2.42 | .83 | .26 | -.17 |
| Child activity | 1.5 | 38705 | 4.02 | .65 | -.49 | -.03 |
|  | 3 | 31611 | 3.62 | .70 | -.12 | -.27 |
|  | 5 | 23354 | 3.22 | .71 | .21 | -.12 |
| Child shyness | 1.5 | 38679 | 2.05 | .64 | .44 | .22 |
|  | 3 | 31600 | 2.22 | .68 | .40 | .17 |
|  | 5 | 23335 | 2.09 | .71 | .43 | .01 |
| Child sociability | 1.5 | 38719 | 3.95 | .56 | -.07 | .16 |
|  | 3 | 31601 | 3.68 | .57 | -.03 | .09 |
|  | 5 | 23353 | 4.07 | .63 | -.24 | -.24 |
| Mother Emotional Symptoms | 1.5 | 37814 | 1.27 | .36 | 2.38 | 8.15 |
|  | 3 | 30617 | 1.26 | .38 | 2.50 | 8.50 |
|  | 5 | 23075 | 1.21 | .33 | 2.79 | 10.82 |

*Child phenotype coding range = 1 – 5. Mother phenotype coding range = 1 – 4. SD = Standard Deviation.*

Table S2. Model fit statistics for within- and cross-generation autoregressive growth-curve models

|  | CFI | TLI | RMSEA (95% CI) | RMSEA p |
| --- | --- | --- | --- | --- |
| Within-generation | | | | |
| Child Emotionality | .998 | .998 | .015 (.001, .021) | 1.00 |
| Child Activity | .993 | .989 | .039 (.034, .045) | .999 |
| Child Shyness | .986 | .979 | .050 (.045, .056) | .454 |
| Child Sociability | .983 | .974 | .041 (.035, .047) | .996 |
| Mother Emotional Symptoms | .998 | .998 | .019 (.013, .025) | 1.00 |
| Cross-generation | | | | |
| Child Emotionality – Mother Emotional Symptoms | .998 | .996 | .014 (.012, .017) | 1.00 |
| Child Shyness – Mother Emotional Symptoms | .993 | .988 | .025 (.023, .028) | 1.00 |
| Child Sociability – Mother Emotional Symptoms | .994 | .989 | .021 (.018, .024) | 1.00 |

*CFI = Comparative Fit Index; TLI = Tucker–Lewis Index; RMSEA = Root Mean Square Error of Approximation; CI = Confidence Intervals; p = probability value*

Table S3. Chi square goodness of fit tests for longitudinal modelling of mother and child variables

| Model | df | AIC | BIC | Chi^2^ | ΔChi^2^ | Δdf | p |
| --- | --- | --- | --- | --- | --- | --- | --- |
| Child Emotionality | | | | | | | |
| 1. Autoregressive growth curve | 2 | 252557.7 | 252618.3 | 21.818 | -- | -- | -- |
| 2. Growth curve | 3 | 252642.0 | 252693.9 | 108.072 | 85.704 | 1 | <.0001 |
| 3. Autoregressive fixed effect | 5 | 252735.4 | 252770.0 | 250.480 | 181.996 | 3 | <.0001 |
| 4. Time-invariant fixed effect | 6 | 252997.6 | 253023.6 | 469.669 | 437.854 | 4 | <.0001 |
| Child Activity | | | | | | | |
| 1. Autoregressive growth curve | 2 | 248670.5 | 248731.1 | 131.481 | -- | -- | -- |
| 2. Growth curve | 3 | 248777.6 | 248829.5 | 240.578 | 109.097 | 1 | <.0001 |
| 3. Autoregressive fixed effect | 5 | 248962.7 | 248997.3 | 429.711 | 298.230 | 3 | <.0001 |
| 4. Time-invariant fixed effect | 6 | 249374.9 | 249400.9 | 843.963 | 712.482 | 4 | <.0001 |
| Child Shyness | | | | | | | |
| 1. Autoregressive growth curve | 2 | 250328.9 | 250389.5 | 216.649 | -- | -- | -- |
| 2. Growth curve | 3 | 250432.1 | 250484.1 | 321.8668 | 105.218 | 1 | <.0001 |
| 3. Autoregressive fixed effect | 5 | 250515.4 | 250550.0 | 409.108 | 192.459 | 3 | <.0001 |
| 4. Time-invariant fixed effect | 6 | 250931.8 | 250957.7 | 827.491 | 610.842 | 4 | <.0001 |
| Child Sociability | | | | | | | |
| 1. Autoregressive growth curve | 2 | 257925.3 | 257985.9 | 143.009 | -- | -- | -- |
| 2. Growth curve | 3 | 257999.0 | 258050.9 | 218.6875 | 75.678 | 1 | <.0001 |
| 3. Autoregressive fixed effect | 5 | 257941.5 | 257976.1 | 165.233 | 22.224 | 3 | .0002 |
| 4. Time-invariant fixed effect | 6 | 258182.2 | 258208.2 | 407.950 | 264.941 | 4 | <.0001 |
| Mother Emotional Symptoms | | | | | | | |
| 1. Autoregressive growth curve | 2 | 241579.4 | 241639.9 | 30.929 | -- | -- | -- |
| 2. Growth curve | 3 | 241631.8 | 241683.7 | 85.355 | 54.427 | 1 | <.0001 |
| 3. Autoregressive fixed effect | 5 | 241764.1 | 241798.7 | 221.645 | 190.716 | 3 | <.0001 |
| 4. Time-invariant fixed effect | 6 | 241869.4 | 241895.3 | 328.901 | 297.972 | 4 | <.0001 |

*df = degrees of freedom; AIC = Akaike Information Criterion; BIC = Bayesian Information Criterion; Chi^2^ = chi-squared test statistic; Δ = change in; p = probability value.*

**Table S4. Goodness of fit tests and parameter estimates for the autoregressive growth curve model (Figure 1, Model 1) fit to within-generation child sociability data: fixing variance in the latent slope factor to zero.** *Note that this is a different approach to dropping the slope latent factor completely (as done in Table S4), which removes slope variance, slope mean, and covariance between slope and intercept. Here we remove slope variance only. Overall, results show evidence for linear change across time, but no individual-level variability in rate of change for sociability.*

1. Chi square goodness of fit tests

|  | **df** | **AIC** | **BIC** | **Chi^2^** | **ΔChi^2^** | **Δdf** | **p** |
| --- | --- | --- | --- | --- | --- | --- | --- |
| Model 1 | 2 | 257925.3 | 257985.9 | 143.009 | -- | -- | -- |
| Model 1,  S variance = 0 | 3 | 257926.9 | 257978.8 | 146.582 | 2.568 | 1 | .109 |

*df = degrees of freedom; AIC = Akaike Information Criterion; BIC = Bayesian Information Criterion; Chi^2^ = chi-squared test statistic; Δ = change in; p = probability value.*

1. Parameter estimates (95% confidence intervals)

|  | Variance in stability: I | Variance in rate of linear change: S | Correlation (*r*):  I – S | Autoregressive effects:  β | Residual error:  e |
| --- | --- | --- | --- | --- | --- |
| Model 1 | .279  (.245, .313) | .005  (-.000, .009) | -.013  (-.019, -.006) | .112  (.085, .140) | .697  (.670, .724) |
| Model 1,  S variance = 0 | .249  (.232, .267) | .000  (.000, .000) | -.007  (-.010, -.004) | .133  (.115, .152) | .721  (.707, .735) |

*I = latent intercept factor; S = latent slope factor*

**Table S5. Chi square goodness of fit tests for nested models in longitudinal analyses of phenotypic, cross-generation data: dropping intergenerational latent factor correlations**

| **Model** | **df** | **AIC** | **BIC** | **Chi^2^** | **ΔChi^2^** | **Δdf** | **p** | |
| --- | --- | --- | --- | --- | --- | --- | --- | --- |
| Base model | | | | | | | |  |
| **Emotionality** | 9 | 492385.6 | 492541.5 | 87.994 | -- | -- | -- | |
| Shyness | 9 | 491797.5 | 491953.4 | 252.895 | -- | -- | -- | |
| Sociability | 9 | 499453.5 | 499609.4 | 175.987 | -- | -- | -- | |
| Drop correlation between Child Intercept – Mother Intercept | | | | | | | |  |
| Emotionality | 10 | 493304.8 | 493452.0 | 1009.152 | 783.237 | 1 | <.0001 | |
| Shyness | 10 | 491839.7 | 491986.9 | 297.100 | 38.561 | 1 | <.0001 | |
| Sociability | 10 | 499462.4 | 499609.6 | 186.890 | 9.722 | 1 | .0018 | |
| Drop correlation between Child Slope – Mother Slope | | | | | | | |  |
| Emotionality | 10 | 492504.3 | 492651.5 | 208.686 | 126.884 | 1 | <.0001 | |
| Shyness | 10 | 491805.7 | 491952.9 | 263.118 | 9.060 | 1 | .0026 | |
| Sociability | 10 | 499453.4 | 499600.6 | 177.886 | 1.708 | 1 | .1912 | |
| Drop correlation between Child Intercept – Mother Slope | | | | | | | |  |
| Emotionality | 10 | 492461.4 | 492608.6 | 165.765 | 59.475 | 1 | <.0001 | |
| Shyness | 10 | 491800.1 | 491947.3 | 257.485 | 4.343 | 1 | .0372 | |
| **Sociability** | 10 | 499451.5 | 499598.7 | 175.992 | .004 | 1 | .9466 | |
| Drop correlation between Child Slope – Mother Intercept | | | | | | | |  |
| Emotionality | 10 | 492387.8 | 492534.9 | 92.111 | 3.55 | 1 | .0594 | |
| **Shyness** | 10 | 491796.1 | 491943.3 | 253.507 | .532 | 1 | .4656 | |
| Sociability | 10 | 499453.5 | 499600.7 | 177.932 | 1.722 | 1 | .1895 | |

*df = degrees of freedom; AIC = Akaike Information Criterion; BIC = Bayesian Information Criterion; Chi^2^ = chi-squared test statistic; Δ = change in; p = probability value. Significance level = .0125 when using Bonferroni correction to account for four multiple tests. Bolded text highlights the best fitting model for each child temperament trait.*

**Table S6. Model fit statistics and comparisons for MCoTS models decomposing the association between stability in mothers’ emotional symptoms and stability in child emotionality**

|  |  |  |  |  | Compare to 1 | | | Compare to 2 | | |
| --- | --- | --- | --- | --- | --- | --- | --- | --- | --- | --- |
|  |  | -2LL | df | AIC | Δ-2LL | Δdf | p | Δ-2LL | Δdf | p |
| 1 | Base model | 130148.0 | 85158 | -40168.03 | -- | -- | -- | -- | -- | -- |
| **2** | **Drop C1 c1’** | **130151.6** | **85160** | **-40168.38** | **3.649** | **2** | **.1613** | **--** | **--** | **--** |
| 3 | Drop C1 c1’ a1’ | 130192.6 | 85161 | -40129.35 | 44.678 | 3 | <.0001 | 41.029 | 1 | <.0001 |
| 4 | Drop C1 c1’ p | 130200.6 | 85161 | -40121.39 | 52.638 | 3 | <.0001 | 48.989 | 1 | <.0001 |

*-2LL = -2loglikelihood; df = degrees of freedom; AIC = Akaike Information Criterion; Δ = change in; p = probability value. Best fitting model indicated in bold.*

Estimates for C1 (indexing variance explained in mothers’ emotional symptoms by shared environmental factors in a mother pair) and c1’ (indexing intergenerational covariance explained by shared environmental factors in the extended family) were not significantly different from zero (Figure S2), so were dropped from the model. This resulted in a more parsimonious model without detriment to model fit (Table S5), thereby increasing statistical power for the estimation of remaining parameters.

**Figure S1. Full MCoTS structural equation model, based around pairs of mothers who are twins, full-siblings, half-siblings, cousins, or unrelated sisters-in-law**

*A1 = additive genetic effects on mother phenotype; C1 = shared environmental effects on mother phenotype; E1 = unique environmental effects on mother phenotype; A2 = additive genetic effects specific to child phenotype; C2 = shared environmental effects specific to child phenotype; E2 = unique environmental effects specific to child phenotype; rA = genetic correlation between relatives; rC = shared environment correlation between relatives; rE = within-mother correlation between E1 for parenting of child 1 and 2. rE is freely estimated to allow for differences in exposure to mother phenotype between siblings born to the same mother. Bold lines show the intergenerational paths used to decompose influence on covariance between parent and child traits: a1’ = genetic effects common to mother and child phenotype; c1’ = extended family shared environment effects*********; p = residual, phenotypic effect between parent and child traits. The pathway between A1 and A1’ is fixed to .50 because parents and children share 50% of their genome. Variance = 1 for all latent factors (omitted for simplicity). For A1’ this means that residual variance (after accounting for the path between A1 and A1’) is .75.*

***** Our definition of the extended family environment (c1’) applies to extended families linked by mothers who are twins, full-siblings or maternal half-siblings. We expect these mothers to have been reared together in the same environment (*r*C=1). We expect that mothers in extended families who are paternal half-siblings, cousins or unrelated sisters/cousins-in-law will not have been reared together in the same environment (*r*C=0), therefore these families do not share an extended family environment.

**Figure S2. Base model results from MCoTS biometric analyses, decomposing the association between stability in mothers’ emotional symptoms and stability in child emotionality**

*A1 = additive genetic effects on mother trait; A2 = additive genetic effects specific to child trait; A1′ = additive genetic effects common to mother and child traits (path from A1 to A1′ is fixed to .50 because offspring inherit 50% of their mother’s genes); C1/C2 = common environment effects on mother/child trait; E1/E2 = unique environment effects on mother/child trait. Path linking C1 to child trait represents extended family shared environment effects (c1’). Variance components are displayed in non-bold text. The residual intergenerational association is displayed as a standardised path beta coefficient in bold text. Figure represents a partial path diagram, see Figure S1 for full model specification.*

1. The term ‘avuncular’ relates to the relationship between individuals and the children of their siblings. For simplicity in this manuscript, we use this term to also include the relationship between individuals and the children of their cousins. [↑](#footnote-ref-1)
